# Clinical feasibility and impact of data-driven respiratory motion compensation studied in 200 whole-body ^18^F-FDG PET/CT scans

**DOI:** 10.1186/s13550-022-00887-x

**Published:** 2022-03-28

**Authors:** André H. Dias, Paul Schleyer, Mikkel H. Vendelbo, Karin Hjorthaug, Lars C. Gormsen, Ole L. Munk

**Affiliations:** 1 Department of Nuclear Medicine and PET Centre, Aarhus University Hospital, Palle Juul-Jensens Boulevard 165, 8200 Aarhus N, Denmark; 2 Siemens Medical Solutions USA, Inc., Malvern, PA USA; 3 Department of Biomedicine, Aarhus University, Aarhus, Denmark; 4 Department of Clinical Medicine, Aarhus University, Aarhus, Denmark

**Keywords:** Gating, Data driven gating, PET, FDG

## Abstract

**Background:**

This study examines the clinical feasibility and impact of implementing a fully automated whole-body PET protocol with data-driven respiratory gating in patients with a broad range of oncological and non-oncological pathologies 592 FDG PET/CT patients were prospectively included. 200 patients with lesions in the torso were selected for further analysis, and ungated (UG), belt gated (BG) and data-driven gating (DDG) images were reconstructed. All images were reconstructed using the same data and without prolonged acquisition time for gated images. Images were quantitatively analysed for lesion uptake and metabolic volume, complemented by a qualitative analysis of visual lesion detection. In addition, the impact of gating on treatment response evaluation was evaluated in 23 patients with malignant lymphoma.

**Results:**

Placement of the belt needed for BG was associated with problems in 27% of the BG scans, whereas no issues were reported using DDG imaging. For lesion quantification, DDG and BG images had significantly greater SUV values and smaller volumes than UG. The physicians reported notable image blurring in 44% of the UG images that was problematic for clinical evaluation in 4.5% of cases.

**Conclusion:**

Respiratory motion compensation using DDG is readily integrated into clinical routine and produce images with more accurate and significantly greater SUV values and smaller metabolic volumes. In our broad cohort of patients, the physicians overwhelmingly preferred gated over ungated images, with a slight preference for DDG images. However, even in patients with malignant disease in the torso, no additional diagnostic information was obtained by the gated images that could not be derived from the ungated images.

**Supplementary Information:**

The online version contains supplementary material available at 10.1186/s13550-022-00887-x.

## Introduction

Positron emission tomography (PET) is well established in the clinical assessment of oncological and infectious/inflammatory diseases [1, 2]. The duration of a clinical whole-body (WB) PET scan is too long to utilize the breath-hold approaches commonly used in computed tomography (CT). Therefore, respiratory motion of organs and lesions are unavoidable during the PET data acquisition resulting in degraded PET image quality due to blurring that effectively lowers the spatial resolution [3]. Motion-induced image degradation may be present from the apex of the lung to the lower abdomen, and particularly near the diaphragm which has been shown to move 8–25 mm during breathing [4]. Such motion is substantial for modern clinical PET imaging in which spatial resolution is 3–5 mm full width at half maximum. Small peri-diaphragmatic lesions may therefore be undetectable in some patients and the measured SUV values may be underestimated. Thus, motion corrected PET imaging could improve lesion detection, characterization, and quantification.

Conventional respiratory gating divides the measured PET data into gates according to the respiratory waveform extracted from external devices, such as a pressure sensitive probe in an elastic belt placed around the patient’s abdomen (Anzai Medical, Tokyo, Japan) or an infrared camera monitoring the motion of a marker placed on the patient’s chest (Varian Medical Systems, Palo Alto, CA, USA). The gated images contain less motion but more noise because they are reconstructed using only a subset of the acquired data. Studies have shown that patients spend most time in the end-expiration quiescent period, and that gated imaging during this phase include the largest fraction of the acquired PET data with minimal motion-induced blurring [5]. Still, a large fraction of PET data is discarded, and a prolonged PET acquisition time is required to compensate for this loss of data. Thus, methods that could use all data would clearly be preferable. Furthermore, the external devices need regular maintenance and calibration, the placement of these external devices on the patients is time consuming, and there is a risk that the external measurements fail during the PET examination.

Data-driven gating (DDG) methods derive the respiratory waveform directly from PET raw data without need of external devices and have been clinically evaluated [6–9]. The magnitude, frequency, phase, and direction of the respiratory motion vary depending on anatomical localization and during the PET examination. Thus, DDG methods tracking the local organ motion directly from the PET data could potentially be more accurate than device-based methods tracking the abdominal surface motion at a single fixed point [7].

A recent study investigated a motion compensation technique that combines a DDG approach with a reconstruction algorithm including a motion deblurring kernel in the iterative loop to generate motion corrected images based on 100% of the acquired data from continuous bed motion (CBM) PET scans [3]. In the present study, we examined the clinical feasibility of this fully automated DDG WB ^18^F-FDG CBM PET examinations in 592 patients. The patients were scanned with a belt with a pressure-sensitive probe, and we reconstructed ungated (UG), belt-gated (BG), and DDG images based on all acquired PET data. In a subset of 200 patients with lesions in torso, we examined the impact of respiratory gating on visual lesion detection, and measured quantitative changes in SUV and metabolic tumour volume evaluation. Finally in 23 patients with malignant lymphoma, one of the few pathologies where small changes in measured SUV can produce clinical consequences, we evaluated whether respiratory gating affects treatment response evaluation based on the Deauville score and Lugano Classification [10].

## Materials and methods

### Patient population

Patients undergoing a clinical ^18^F-flurodeoxyglucose (FDG) PET/CT at our department between February and October 2020 (full population, *N* = 592) were prospectively included. All included patients were informed of the study purpose and a written informed consent was obtained before participation. Approval for the study was obtained from the regional ethics committee (1-10-72-194-19).

An experienced physician (AHD) reviewed all images to select a subset of patients with FDG-avid lesions in the torso region, which is most affected by respiratory motion (image evaluation group, *N* = 200). In addition, a smaller subset of lymphoma patients with lesions in the torso region were identified (lymphoma group, *N* = 23).

Encompassing a random sample of the clinical population at our department, the subset of patients scanned covered a varied sample of oncological and non-oncological pathologies. A detailed listing of scan indications for referral to the department as well as demographics for the study populations are displayed in Additional file 1: Table S1 (full population) and Table 1 (image evaluation group).

**Table 1:** Scan indications and demographic distribution of image evaluation group

Scan indication	Number of patients			Age distribution*			BMI*		
	Total	Male	Female	All	Male	Female	All	Male	Female
Breast Cancer	1	–	1	51	–	51	28.4	–	28.4
Cancer of unknown primary origin	3	1	2	68	87	58 [57–59]	29.7 [22.2–37.6]	22.2	33.65 [29.7–37.6]
Gastro–intestinal cancer	21	17	4	67 [29–83]	67 [29–83]	64 [44–75]	26.2 [16.8–40.6]	26.5 [16.8–40.6]	20.8 [18.9–27.6]
Head & Neck cancer	12	8	4	64.5 [32–81]	66 [53–81]	62.5 [32–66]	24.6 [22.6–37.4]	25.7 [22.8–36.5]	22.9 [22.6–37.4]
Infection & Inflammation	13	8	5	64 [21–89]	58.5 [27–78]	64 [21–89]	24.2 [20.7–36.8]	23.9 [20.7–36.8]	24.6 [21.9–26.4]
Lung Cancer	97	52	45	74 [42–89]	75 [42–86]	72 [42–89]	25.8 [14.5–44.4]	26 [14.5–42.6]	25.7 [16–44.4]
Lymphoma	23	14	9	67 [18–80]	62 [39–78]	67 [18–80]	25.3 [19.4–45.4]	27.2 [19.8–45.4]	23.7 [19.4–31.6]
Melanoma	10	5	5	62.5 [52–81]	64 [57–79]	60 [52–81]	27 [23.1–31.3]	27.1 [25.5–28.7]	26.8 [23.1–31.3]
NET	1	1	–	68	68	–	25.5	25.2	–
Sarcoma	1	–	1	63	–	63	32.5	–	32.5
Uro–genital cancer	18	2	16	65 [26–87]	63 [59–67]	66 [26–87]	25.8 [20.5–47.7]	33.5 [32.2–34.7]	24.2 [20.5–47.7]
Total	200	108	92	68 [18–89]	70 [27–87]	70 [18–89]	25.6 [14.5–47.7]	26.1 [14.5–45.4]	25.4 [16–47.7]

*Values are median [range]

### Data acquisition and image reconstruction

PET/CT data were acquired using our standard oncological scan protocol on a Siemens Biograph Vision 600 (Siemens Healthineers) with a 26.2 cm axial PET field-of-view. Patients were intravenously injected with ^18^F-FDG (4 MBq/kg), rested for the 60-min uptake period, and were placed in the scanner in the supine position with arms above the head. A trained technician carefully instructed the patients and placed an elastic belt with a pressure-sensitive probe (AZ-733 V, Anzai Medical Corporation) around the abdomen. A low dose WB CT (25 Ref mAs, 120 kV, CARE Dose4D, CARE kV, ADMIRE level 3) was performed, followed by a WB CBM PET scan (1.5 mm/s). All PET images were reconstructed using TrueX + TOF, 4 iterations / 5 subsets, 440 matrix, 2 mm Gaussian post filter. The cubic voxel size was 1.65^3^ mm^3^, and the central spatial resolution was around 3.3 mm in full width at half maximum. Three PET images were reconstructed from the same data using the same acquisition time for all images: an UG image without motion correction, an image with BG respiratory gating and motion correction (OncoFreeze, Siemens Healthineers), and an image with DDG respiratory gating and motion correction (OncoFreeze AI, Siemens Healthineers). The first two images were reconstructed on the scanner with software version VG76B, whereas the latter was reconstructed on a research workstation with a beta version of VG80 software.

The respiratory motion compensation was employed for the torso part of the image from upper lung to below the liver. Essentially, the difference between the motion compensated images is determined by the method by which the respiratory waveform in generated; BG uses the signal generated by the external Anzai belt with a pressure-sensitive probe, whereas DDG derives the waveform directly from the acquired CBM PET raw data [3, 7]. For both methods, optimal gate images are reconstructed using the default 35% of the PET data with the lowest amplitude corresponding to an approximately motion-free image in end-expiration [11]. A motion blurring kernel, that maps the optimal gate image into the ungated PET image, is estimated using a mass conservation-based optical flow method [12, 13]. The motion-blurring kernel is used in the forward projection step of the iterative reconstruction methods to allow reconstruction of motion compensated images using 100% of the acquired PET data [14].

### Clinical feasibility of PET scanning using belt-gated respiratory gating

For the full population (*N* = 592 patients), trained technologists instructed the patient prior to the scans, carefully placed and tightened the belt around the abdominal part with most respiratory motion, and verified both on the display on the scanner and on the Anzai Sensor Port that a clear respiratory waveform was registered. A clinical feasibility survey was filled out by the technologists acquiring the images. The technologists recorded patient demographic data while also registering any problems related to the placement of the belt, the quality of the respiratory waveforms, and reconstruction of BG images.

### Semi-quantitative image analysis

For the image evaluation subgroup (*N* = 200 patients), an experienced nuclear medicine physician (AHD) visually identified the “most blurry” lesion on the UG images, i.e., a representative lesion clearly affected by motion-induced blurring, and delimited this lesion in a volume of interest (VOI). This VOI was then adjusted to the contours of the lesion with the isocontour tool in PMOD 4.0 (PMOD Technologies Ltd) using the three most commonly used delimitation thresholds: SUV > 2.5, SUV > 41% SUV_max_, and SUV > 50% SUV_max_. In addition, a reference area in the liver was delimited by drawing a spherical VOI with a radius = 20 mm (volume = 33.5 mL) in an area of hepatic tissue devoid of pathological findings. This method was applied for the 3 gating reconstructions (UG, BG and DDG), and measurements of SUV (mean, max, SD) and metabolic volume were registered.

### Visual image analysis and clinical evaluation

For the image evaluation subgroup (*N* = 200), patient data were anonymized and the three PET images (UG, BG, DDG) were assigned a random letter (A, B, C) and sent to the workstations that the physicians use in daily clinical practice when reading PET/CT examinations. Three experienced nuclear medicine physicians (KH, MV, LCG) analysed each dataset in Hermes Gold Client v.2.5.0 (Hermes Medical Solutions AB). A custom display application was created mimicking the display window used for daily clinical practice, but with the three reconstructions displayed side-by-side for easy comparison.

The 3 physicians participating in the blinded study evaluated UG, BG, and DDG images of 200 patients, i.e. the information obtained from this image evaluation included 1800 individual assessments. The evaluations were done individually and with no communication between the physicians. As part of the visual image evaluation, the physicians filled out a survey regarding image quality (classifying the scans as “unusable”, “low quality”, “acceptable quality”, “good quality”), the presence of motion blurring or artefacts, preference in terms of image quality and readability, and presence of additional clinically relevant information in any of the scans.

### Evaluation of lymphoma patients using the Deauville criteria

For the lymphoma subgroup (*N* = 23), the Deauville scoring system is used in clinical routine for treatment response assessment as advocated by international guidelines [10]. The Deauville scoring system is a visual comparison of lymphoma residual lesion SUV_max_ with the SUV_max_ of the liver and blood pool.

**Fig. 1:**
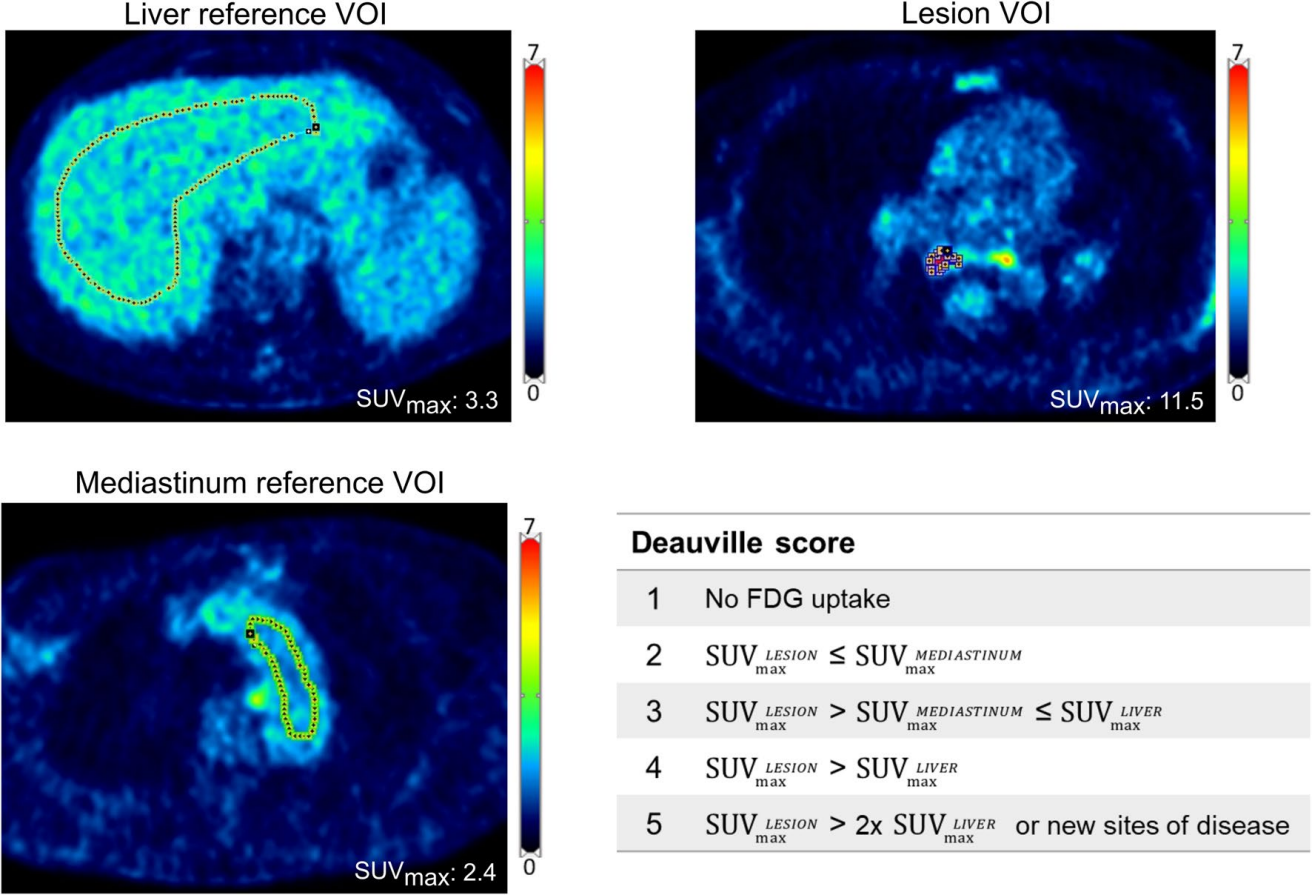
Example of VOI delimitations used for the Deauville scoring. This patient with a T-cell lymphoma displayed multiple new lesions in the thorax area. Deauville score = 5

VOIs supporting the visual estimations were delimitated by the method used in daily clinical routine at our department (see Fig. 1). The most representative area of disease was measured as described in the semi-quantitative analysis section. A VOI of the background liver activity was delineated by drawing a large “banana-shaped” ROI in 3 consecutive slices, whilst the background activity in the mediastinum was obtained by delineating an oblong ROI in the lumen of the aortic arch in 2 consecutive slices, taking care not to include activity from the vascular wall [15]. SUV_max_ was extracted for the lesion and both background areas and target-to background ratios with corresponding Deauville scores were then calculated for each of the 3 gating reconstructions.

### Statistical analysis

The statistical and graphical analysis of the extracted data was performed using Stata 16 and Graphpad Prism 9.1.0. The Anderson–Darling Test and Kolmogorov–Smirnov Test were performed in Graphpad to assess the Gaussian distributions of the data sets. Statistical *T*-tests of unpaired non-parametric data (BMI values) were performed using the Mann–Whitney test. Statistical *T*-tests of paired parametric data (Liver SUV_max_, Liver SUV_mean_ and CoV values) were performed using paired *T*-tests. Statistical tests of paired non-parametric data (Lesion SUV_max_, Lesion SUV_mean_ and metabolic volume) were performed using the Wilcoxon test. *P*-values of < 0.05 were considered significant. Inter-user agreement was evaluated by Fleiss-Kappa and rated according to the classification proposed by Altman, 1991 [16]. Bland–Altman analysis was performed by plotting of differences between method A and method B, expressed as percentages of the values on the axis [(Method A–Method B) / mean)], *vs*. the mean of the two measurements.

## Results

### Clinical feasibility

Patients were described as very collaborative by the nuclear medicine technologists in 90% of cases (530/592), 59 patients were described as moderately collaborative and only 3 of the patients in the study were described as uncollaborative. However, the Anzai belt was considered “moderate” to “difficult” to place in 27% of patients (159/592) independent of the patients’ body mass. The average BMI for the “easy placement” group was 26.3 ± 5.0 kg/m^2^, while the average BMI for the “moderate to difficult placement” group was 27.0 ± 5.5 kg/m^2^ (*p* = 0.19). On average, experienced technologists spent an additional 72 s (range [40 s–165 s]) placing the belt.

### Semi-quantitative analysis

**Fig. 2:**
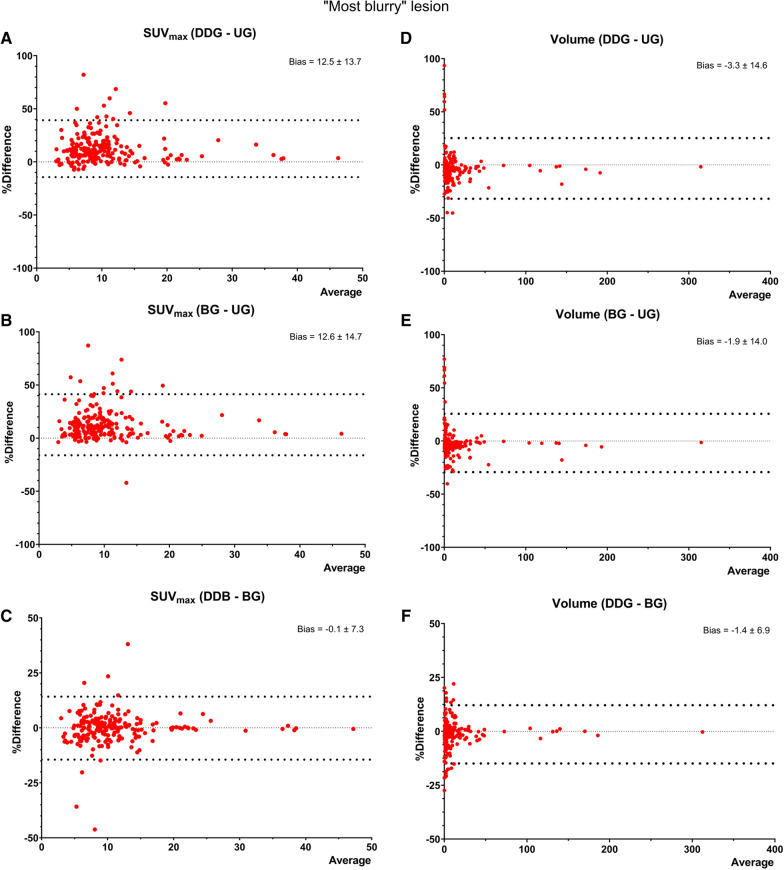
Bland–Altman plots of the “most blurry” lesion (*N* = 200), representing the differences between SUV_max_ values (**A**–**C**), and differences between metabolic volumes (**D**–**F**). Note: For the smallest lesions, very high volume differences (in %) can be caused by the inclusion/exclusion of few voxels

When using a VOI delineation threshold of SUV > 2.5 (Table 2), the analysis of the “most-blurry” lesion showed an overall slight increase (~ 8%) in the measured median SUV_mean_ between the ungated and gated reconstructions. We observed a statistically significant difference between the ungated and gated reconstructions (*p* < 0.0001) and between gated reconstructions (p = 0.02). SUV_max_ values showed the same tendency (increase of ~ 14%), with statistically significant difference between the ungated and gated reconstructions (*p* < 0.0001), but no statistically significant difference between BG and DDG. A visual representation of the percentual difference between SUV_max_ values is displayed in Fig. 2, and for SUV_mean_ values in Additional file 1: Figure S1. Motion correction methods led to an overall reduction (~ 12%) in the metabolic volume of the analysed lesions, with statistically significant differences between all 3 reconstructions, with the biggest volume reduction for DDG.

**Table 2:** Characteristics of all 200 “most blurry” lesions for all reconstructions

Lesion	UG	BG	DDG
SUV_max_	8.29 [2.86–45.4]	9.50 [2.88–47.3]	9.43 [3.01–47.1]
*P* value to UG		< 0.0001	< 0.0001
*P* value to BG			0.98
SUV_mean_	3.77 [1.19–12.8]	4.04 [1.2–13]	4.07 [1.19–12.9]
*P* value to UG		< 0.0001	< 0.0001
*P* value to BG			0.02
Metabolic volume (mL)	4.28 [0.03–317]	3.81 [0.05–313]	3.77 [0.05–312]
*P* value to UG		< 0.0001	< 0.0001
*P* value to BG			0.006

SUV values and Metabolic volumes are median [range]

The same tendencies were observed when using a VOI delineation threshold of SUV > 41% SUV_max_, and SUV > 50% SUV_max_, although without statistically significant differences between the two gating reconstructions (see Additional file 1: Table S2).

**Fig. 3:**
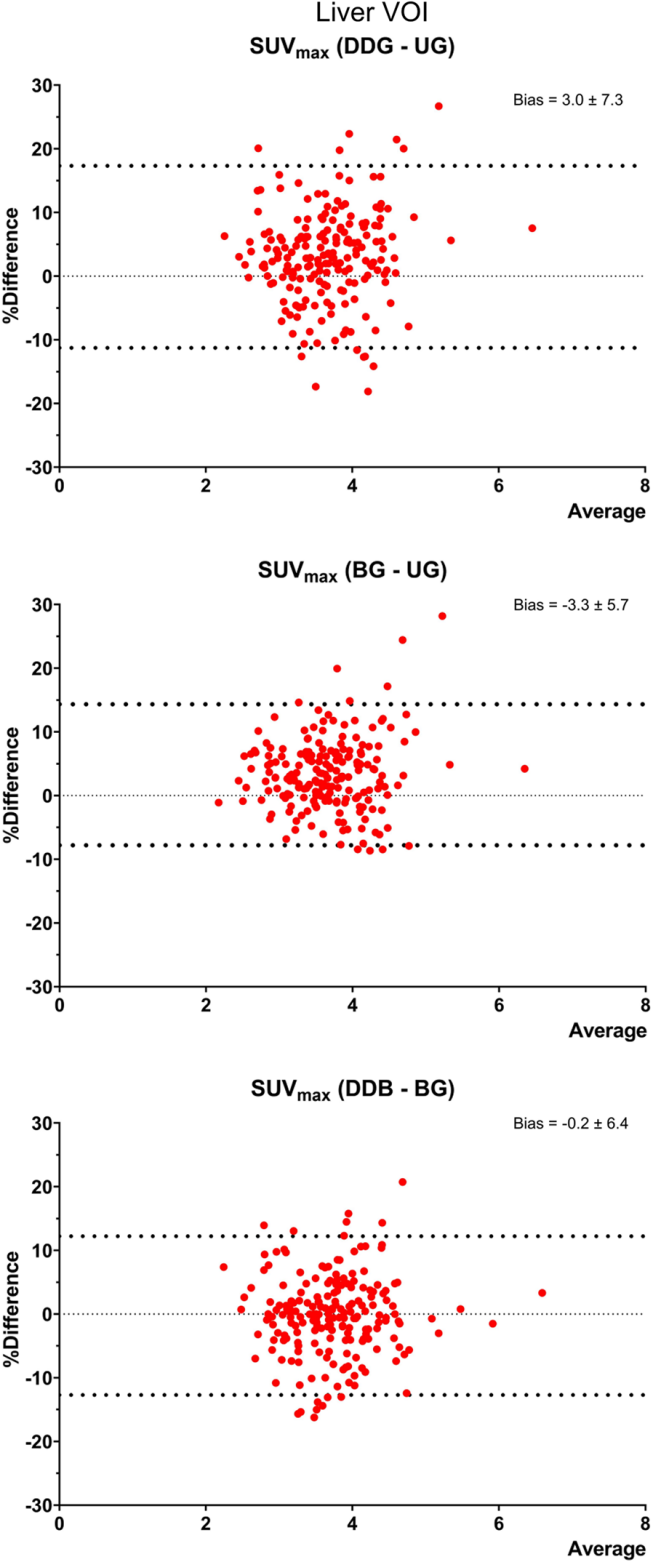
Bland–Altman plots for the fixed liver VOI, representing the differences between SUV_max_ values. *N* = 199 (one patient excluded due to artefact in liver region, see Fig. 4)

Compared to the UG reconstruction, the reference liver region (Fig. 3) showed a small but significant increase (~ 4%) SUV_max_ and decrease (~ 1%) in SUV_mean_ (Additional file 1: Figure S2) using two gated reconstructions (Table 3). In addition, the coefficient of variation (CoV) in the two gated reconstructions was slightly but significantly higher than in the UG reconstruction [CoV (%): UG = 15.1 ± 1.9; BG = 16.7 ± 2.0; DDG = 16.6 ± 2.1], which is a measure often used to evaluate image noise. No significant differences between BG and DDG were observed.

**Table 3:** Characteristics of a fixed liver VOI for all reconstructions

Liver	UG	BG	DDG
SUV_max_	3.59 [2.18–6.21]	3.71 [2.16–6.48]	3.73 [2.33–4.38]
*P* value to UG		< 0.0001	< 0.0001
*P* value to BG			0.91
SUV_mean_	2.23 [1.33–2.94]	2.21 [1.33–2.94]	2.21 [1.33–3.08]
*P* value to UG		< 0.0001	< 0.0001
*P* value to BG			0.64

SUV values are median [range]

### Qualitative analysis

**Fig. 4:**
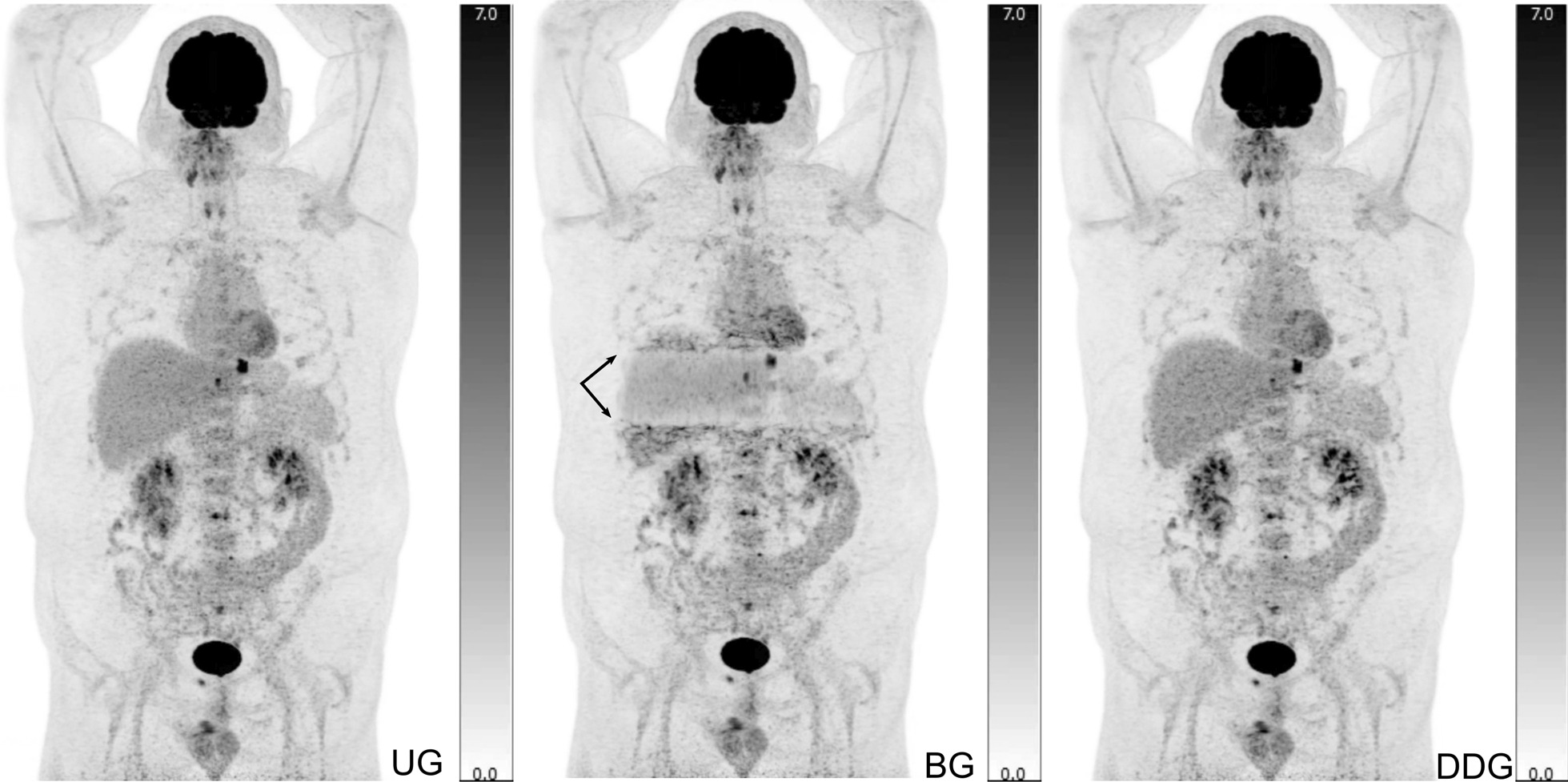
This 48-year-old man was scanned as part of lymphoma evaluation. The belt-based gating (BG) reconstruction introduced a “band” artefact (arrows) over the liver region, which hindered clinical evaluation

The physicians’ assessment of the 200 patients entailed the individual and side-by-side evaluation of 600 reconstructions. Images were overwhelmingly reported as retaining diagnostic quality by all readers (kappa = 1). Only a single BG reconstruction was unusable due to a large reconstruction artefact, as displayed in Fig. 4. Using a four-level scale to characterize the visual quality of these images, only < 1% of reconstructions were considered in the lowest categories of “unusable” or “low quality” (11/1800 assessments). Even the ungated reconstructions were predominantly rated as of “acceptable quality” (127/600) or “good quality” (466/600).

**Fig. 5:**
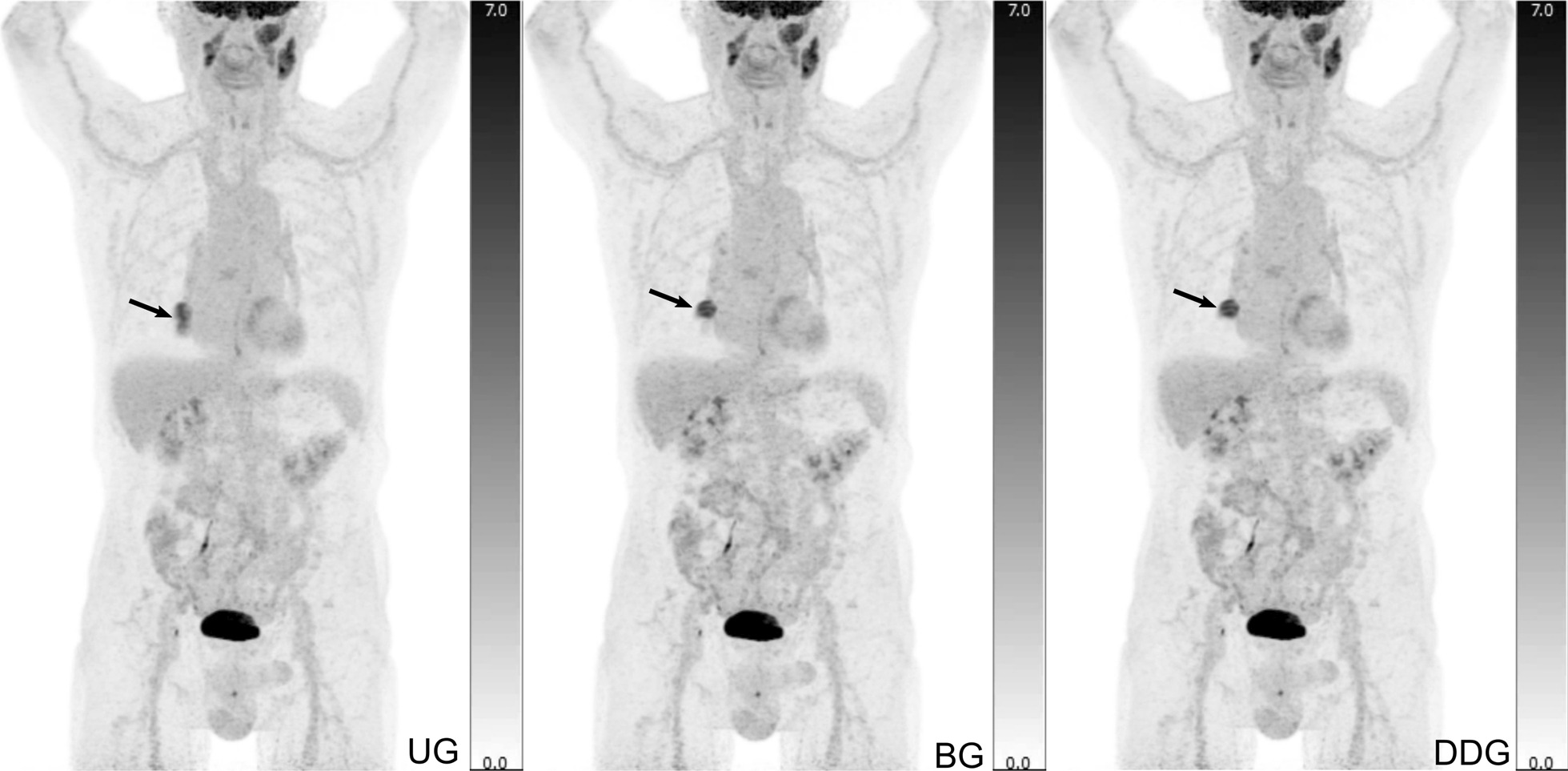
A 79-year-old male patient with lung cancer displaying a classic motion artefact. The ungated image (UG) appears to have a double contouring of the primary tumour (arrows), whereas the two motion compensated images (BG, DDG) clearly reveal a single lesion with sharper contours, smaller metabolic volume and increase in SUV values

In roughly half of the PET examinations, patient movement and respiratory motion led to image artefacts. Notable blurring and movement artefacts were reported by a physician in 44% (87/200) of the patients, although with only fair inter-rater agreement (kappa = 0.24). An example of such blurring is displayed in Fig. 5. The blurring artefacts were mostly found only in UG images (62/200) but in 25 patients also in the motion compensated images: UG + BG + DDG (17/200), UG + BG (5/200), and UG + DDG (3/200). Thus, respiratory motion compensation did not always remove blurring artefacts completely leaving some blurring artefacts that could be caused by insufficient respiratory motion compensation within the gated volume, motion outside the gated volume, or bulk motion that is unrelated to respiration. In 4.5% (9/200) of the UG images, a physician reported that severe blurring was a problem for clinical image evaluation, whereas this was never reported for BG and DDG images. This analysis showed fair inter-rater agreement (kappa = 0.26).

In 2% (4/200) of the patients, the physicians reported uncertainty regarding the anatomical localization of a certain lesion, most often related to whether a lesion was located in the lung or the liver. The uncertainty was reported in UG only (2/200) and in UG + BG + DDG (2/200), which indicates that motion compensation reduced this problem. This analysis showed poor inter-rater agreement (kappa = 0.20).

Overall, when asked which of the reconstructions was preferred, the physicians chose a gated reconstruction 97% (582/600) of times. There was a slight preference towards the DDG reconstructions (301/582) over the BG reconstructions (281/582). When given the task of blindly identifying the ungated reconstruction, the physicians were able to do so in 94% of datasets (566/600), with very good inter-evaluator agreement (kappa = 0.88).

Finally, in 4.5% of cases (9/200) a physician noted that one of the gated images yielded apparent additional diagnostic information. These findings were mostly of uncertain aetiology and showed poor inter-rater agreement (kappa = 0.17). Follow up imaging studies (CT or control PET/CT) were unable to confirm any of these “additional” reported findings.

### Evaluation of lymphoma patients using the Deauville criteria

**Fig. 6:**
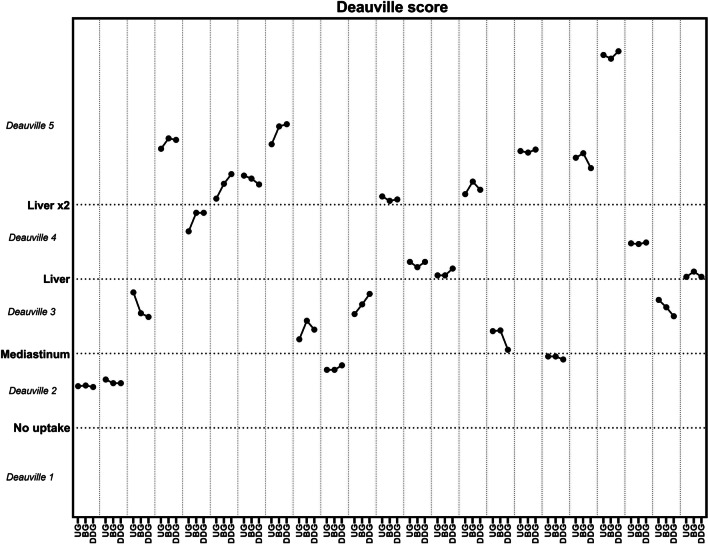
Changes in target-to-background ratios (TBR’s) and corresponding Deauville scores using the 3 different gating reconstructions. Deauville scores are reported as a range on a continuous TBR scale as SUV_max_ in the lesion divided by SUV_max_ in reference tissue (liver and/or mediastinum)

The analysis of a subpopulation of 23 lymphoma patients showed the same clinical Deauville score for all reconstruction methods (Fig. 6). However, changes in SUV_max_ values were observed not only for the residual lesions, but also for the reference liver and mediastinal blood pool VOIs, on BG and DDG images. Therefore, motion compensation could indeed cause a change in Deauville score.

## Discussion

Image blurring and poor PET/CT co-registration in the torso has been a perceived clinical problem since PET/CT gained widespread use in diagnostic imaging [1, 17]. Consequently, various respiratory gating techniques have been developed to improve both modality co-registration and correct assessment of tissue and lesion radioactivity concentration. In this study, we therefore compared UG images with DDG and BG corrected images to determine whether respiratory gating in a clinical setting improves or alters lesion detection, lesion quantification and treatment response evaluation.

### Visual impact of respiratory gating

Lesion blurring depends on the patient’s respiratory movement and the anatomical localization of the lesion. Unsurprisingly, some patients have modest respiratory motion resulting in images that are almost unaffected by motion artefacts. However, in 94% of our cases reading physicians could identify the UG image, and in 97% the physicians selected a gated image as their preferred image. Thus, almost all images were affected by image blurring in our cohort of patients with pathology in the torso.

For this visual image analysis, the physicians evaluated the full UG, BG and DDG volumes using the department’s standard image evaluation tools, whereas the visual scoring in some studies have prepared specific slices through pre-identified lesions [3, 6]. The complete image analysis therefore allowed us to evaluate whether respiratory motion compensation led to identification of more lesions. One should note that the visual evaluation of image quality is fundamentally a subjective process. In our study, three independent raters were invited to use their personal experience to perform this qualitative analysis, which resulted in low inter-observer agreement in a few categories. Overall, we consider this to reflect the subjective nature of visual analysis and interpretation of clinical images, particularly on the subject of blurring. Lesion blurring in UG images was reported as notable in 44% of the cases but only as problematic for clinical evaluation in 4.5% of the cases. Even though the physicians overwhelmingly preferred gated images, only in 4.5% of all cases did the physicians note that a gated image yielded additional (but unconfirmed) diagnostic information with poor inter-rater agreement. Thus, whereas motion compensated images clearly facilitate image evaluation, we did not find a convincing clinical impact of BG or DDG based on visual evaluation in our patient cohort with a broad range of indications (Table 1).

Contrasting this, it was recently reported by another group that DDG may detect 24% more lesions and result in management change in 8% of patients [18]. It is possible that this discrepancy is caused by different criteria used to define a lesion as “new” and also by the use of a PET/CT protocol where the DDG method prolongs the scan time by 4:37 min on average, and up to 10 min in some patients.

### Quantitative impact of respiratory gating and impact on treatment response assessment

Our results were based on patients from our typical clinical patient cohort, and images were reconstructed and reviewed using our standard methods. Overall, respiratory gating had the anticipated effect on radiotracer quantitation, since SUV_max_ values in lesions increased by 14%, whereas lesion volumes decreased by roughly the same (Table 2). These changes are somewhat smaller than the reported changes (~ 40%) from a comparable study using the same gating method [3]. In that study, patients were uniformly diagnosed with a malignant disease and images were reconstructed with a focus on high resolution and no filtering. Image quantification is strongly affected by the choice of image reconstruction parameters, and image reconstruction with smaller voxel size and without post-filtering results in images with higher spatial resolution, where motion compensation impact more on SUV and volume measures [3]. However, some filtering is usually applied to clinical images and we therefore believe that our results better represent the performance of DDG in daily clinical practice.

We found substantial and statistically significant differences between SUV_max_, SUV_mean_ and volume measured on ungated and gated images (Table 2). The pairwise statistical analyses also led to statistically significant higher tumor SUV_mean_ and lower volume in the DDG images compared with the BG images, whereas this was not the case for the inherently noisier SUV_max_. This would suggest that DDG compensate slightly better for motion than BG, but the absolute differences between BG and DDG were small and unlikely to be clinically relevant. For most clinical purposes, a 10% change in SUV_max_ or lesion volume is unlikely to matter much, and although some attempts have been made at standardizing treatment response evaluation in solid tumours through measurements of SUV values (PERCIST 1.0 [19]), these criteria have not gained widespread use. However, in malignant lymphoma, even discrete alterations in measured SUV values may not be trivial. Lymphoma treatment response assessment is based on the Deauville Score, in which the metabolic activity of residual lymphoma masses is graded on a 5-point scale depending on the lesion-to-reference tissue ratio [20]. Any residual lymphoma mass with visual FDG uptake above activity in the liver is considered indicative of active disease as outlined in the Lugano classification [10], and warrants either increased surveillance, consolidative radiotherapy or escalation of chemotherapy. The Deauville Score was developed as a purely visual grading of lesion-to-background FDG uptake, but is often substantiated by measuring the ratio between lymphoma SUV_max_ and SUV_max_ in a representative VOI in the liver [15]. Even modest increases in lesion and liver SUV_max_ values may therefore in theory result in a changed lymphoma/liver ratio and consequently in a changed treatment response assessment. As seen in Fig. 6, respiratory gating did change several lymphoma/liver ratios quite considerably even though no alterations in DS were observed. In our opinion, respiratory gating should therefore always be performed during lymphoma treatment response PET/CT, simply to eliminate the risk of Lugano misclassification.

## Conclusion

Respiratory motion compensation techniques allow superior FDG PET image quality without causing prolonged scan time by using 100% of the acquired PET data. DDG-based imaging can be readily integrated in the clinical routine, images are rapidly reconstructed and DDG has proven to be a fail-safe and robust alternative to BG. For visual analysis, reading physicians overwhelmingly preferred motion compensated images with a slight preference for DDG images. For lesion quantification, DDG and BG images had significantly greater SUV values and smaller volumes than UG images, which could have an impact on risk stratification by metabolic tumour volume assessment. However, in a broad cohort of patients with lesions in the torso, no additional lesions were detected by applying either gating method, and lymphoma treatment response evaluation by the Deauville Score was also unaffected by applying gating to the images.

## Supplementary Information

**Additional file 1**. **Table S1**: Scan indications and demographic distribution of full patient population. **Table S2**: Characteristics of all 200 “most blurry” lesions for all reconstructions using 3 different thresholds for isocontouring. **Figure S1**: Bland–Altman plots of the “most blurry” lesion (*N* = 200), representing the differences between SUVmean values. **Figure S2**: Bland–Altman plots for the fixed liver VOI, representing the differences between SUVmean values.

## Data Availability

Within the restrictions applied by the EU GDPR, all data are available from the authors upon reasonable request.

## Abbreviations

BG: Belt gated
BMI: Body mass index
CBM: Continuous bed motion
CT: Computed tomography
DDG: Data-driven gating
FDG: ^18^F-flurodeoxyglucose
PET: Positron emission tomography
SUV: Standardized uptake value
UG: Ungated
WB: Whole-Body
