# Supplementary material for: Clinical feasibility and impact of data-driven respiratory motion compensation studied in 200 whole-body 18F-FDG PET/CT scans
Source: EJNMMI Res. 2022 Mar 28;12:16. doi: 10.1186/s13550-022-00887-x (PMC8960547; doi:10.1186/s13550-022-00887-x)
Supplement: Supplementary file 1 — Additional file 1. Table S1: Scan indications and demographic distribution of full patient population. Table S2: Characteristics of all 200 “most blurry” lesions for all reconstructions using 3 different thresholds for isocontouring. Figure S1: Bland–Altman plots of the “most blurry” lesion (N = 200), representing the differences between SUVmean values. Figure S2: Bland–Altman plots for the fixed liver VOI, representing the differences between SUVmean values. [file 13550_2022_887_MOESM1_ESM.pdf]

Clinical feasibility and impact of data-driven respiratory motion compensation studied in 592 whole-body ^{18}F-FDG PET/CT scans

André H. Dias[1], Paul Schleyer[2], Mikkel H. Vendelbo[1,3], Karin Hjorthaug[1], Lars C. Gormsen[1,4], Ole L. Munk[1,4]

1 Department of Nuclear Medicine and PET Centre, Aarhus University Hospital, Denmark; 2 Siemens Medical Solutions USA, Inc.; 3 Department of Biomedicine, Aarhus University; 4 Department of Clinical Medicine, Aarhus University

Corresponding author: André H. Dias (andre.dias@auh.rm.dk)

Supplemental material:

Table 1: Scan indications and demographic distribution of full patient population

Scan indication	Number of patients			Age distribution*			BMI*		
	**Total**	**Male**	**Female**	**All**	**Male**	**Female**	**All**	**Male**	**Female**
Breast Cancer	1	-	1	51	-	51	28.4	-	28.4
Cancer of unknown primary origin	13	4	9	63 [29-87]	65.5 [61-87]	59 [29-76]	26.4 [20.5-37.6]	23.3 [22.2-27.5]	26.8 [20.5-37.6]
Gastro-intestinal cancer	31	25	6	68 [29-83]	68 [29-83]	63 [44-75]	26.2 [16.8-40.6]	26.5 [16.8-40.6]	22.8 [18.9-27.6]
Head & Neck cancer	31	16	15	65 [32-98]	64.5 [53-81]	65 [32-98]	23.9 [19.3-37.4]	24.1 [20.5-36.5]	23.7 [19.3-37.4]
Infection & Inflammation	54	30	24	67.5 [21-89]	60.5 [27-81]	68 [21-89]	26.7 [19-39.1]	26.7 [20.7-36.8]	25.5 [19-39.1]
Lung Cancer	160	89	71	73 [36-89]	74 [42-86]	72 [36-89]	25.5 [14.5-44.4]	26 [14.5-42.6]	24.2 [16-44.4]
Lymphoma	68	42	26	66 [18-85]	67 [18-85]	63 [18-80]	25.4 [19.4-45.4]	26.2 [19.8-45.4]	23.8 [19.4-38]
Melanoma	130	61	69	63 [25-92]	66 [25-88]	62 [27-92]	26.4 [17.6-38.6]	27.4 [21.8-38.6]	24.8 [17.6-36.8]
NET	2	1	1	72 [68-76]	68	76	27.3 [25.2-29.4]	25.2	29.4
Sarcoma	14	6	8	54 [25-84]	52 [25-70]	58 [40-84]	28 [19.8-40.4]	24.3 [20.6-33]	30.9 [19.8-40.4]
Uro-genital cancer	88	23	65	67 [26-88]	72 [37-85]	65 [26-88]	25.9 [17.9-50.2]	26.9 [19-34.7]	25.2 [17.9-50.2]
**Total**	592	297	295	68 [18-98]	69 [18-88]	67 [18-98]	25.6 [14.5-50.2]	26.3 [14.5-45.4]	24.8 [16-50.2]

*. Values presented as Median [range]

Table 2: Characteristics of all 200 "most blurry" lesions for all reconstructions using 3 different thresholds for isocontouring

	Threshold SUV2.5			SUV41%max			SUV50%max		
	**UG**	**BG**	**DDG**	**UG**	**BG**	**DDG**	**UG**	**BG**	**DDG**
SUV$_{max}$	8.29	9.50	9.43	8.29	9.50	9.43	8.29	9.50	9.43
	[2.86-45.4]	[2.88-47.3]	[3.01-47.1]	[2.86-45.4]	[2.88-47.3]	[3.01-47.1]	[2.86-45.4]	[2.88-47.3]	[3.01-47.1]
P value to UG		<0.0001	<0.0001		<0.0001	<0.0001		<0.0001	<0.0001
P value to BG			0.98			0.996			0.87
SUV$_{mean}$	3.77	4.04	4.07	4.68	5.55	5.59	5.34	6.28	6.22
	[1.19-12.8]	[1.2-13]	[1.19-12.9]	[1.54-26.4]	[1.64-27.0]	[1.64-27.0]	[1.75-28.4]	[1.83-29.1]	[1.37-29.0]
P value to UG		<0.0001	<0.0001		<0.0001	<0.0001		<0.0001	<0.0001
P value to BG			0.02			0.45			0.95
Volume (mL)	4.28	3.81	3.77	2.61	1.72	1.72	1.26	0.97	0.95
	[0.03-317]	[0.05-313]	[0.05-312]	[0.13-132]	[0.10-114]	[0.09-113]	[0.09-99.9]	[0.07-73.8]	[0.07-81.6]
P value to UG		<0.0001	<0.0001		<0.0001	<0.0001		<0.0001	<0.0001
P value to BG			0.006			0.46			0.64

SUV values and Metabolic volumes are median [range]

UG: Ungated; BG: Belt-gating; DDG: Data-driven gating

"Most blurry" lesion

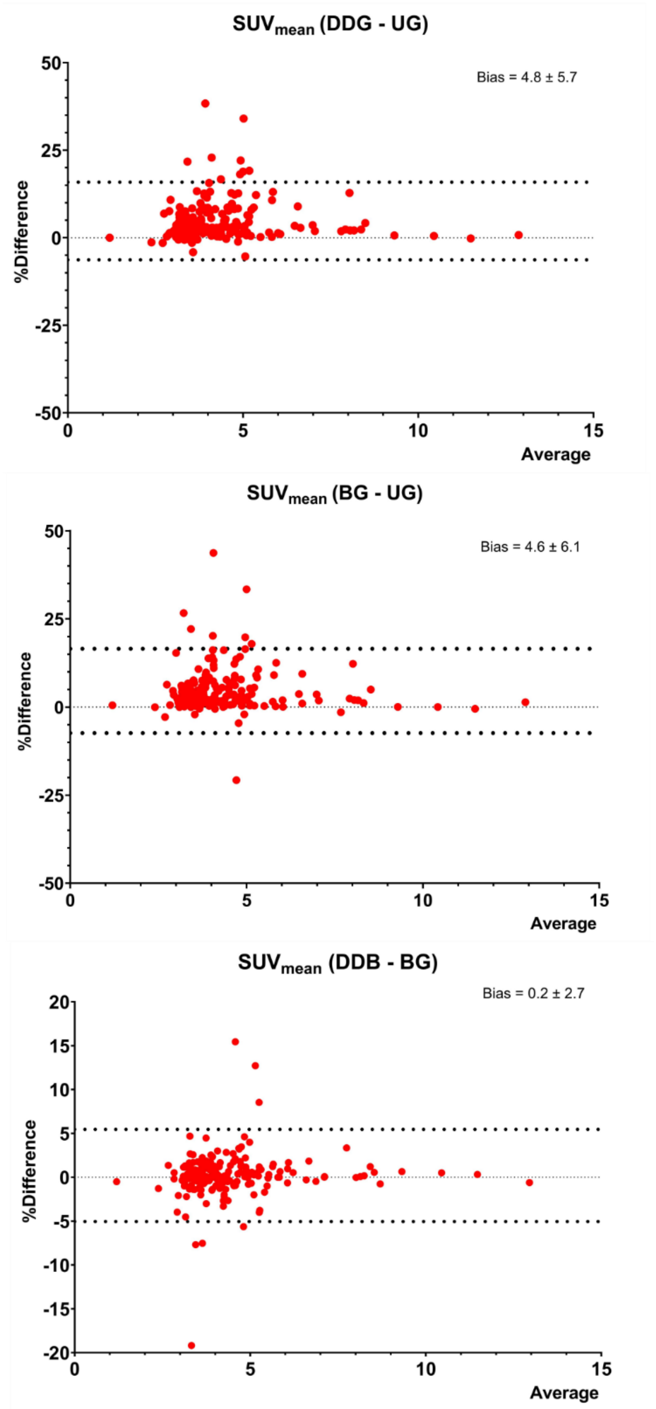

FIGURE.1. Bland-Altman plots of the "most blurry" lesion (N=200), representing the differences between SUV_{mean} values.

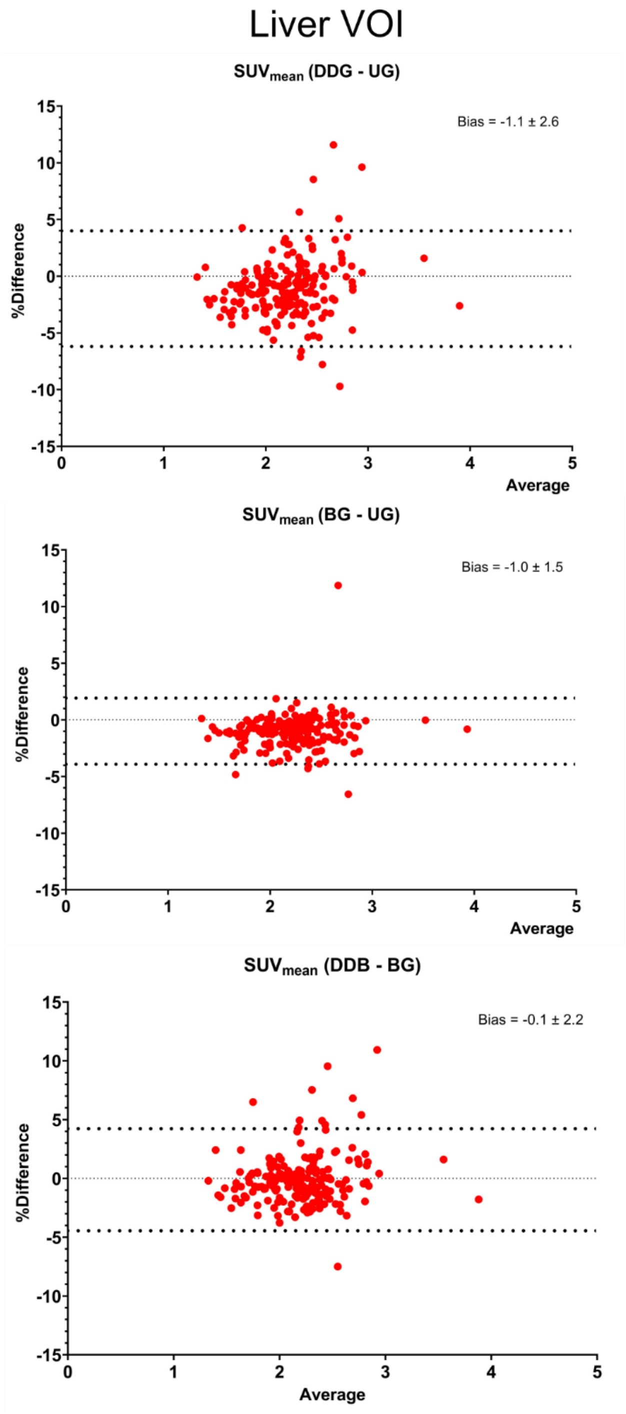

FIGURE.2. Bland-Altman plots for the fixed liver VOI, representing the differences between SUV_{mean} values. N=199 (one patient excluded due to artefact in liver region, see manuscript figure 4)
